# Leveraging Bayesian networks and information theory to learn risk factors for breast cancer metastasis

**DOI:** 10.1186/s12859-020-03638-8

**Published:** 2020-07-10

**Authors:** Xia Jiang, Alan Wells, Adam Brufsky, Darshan Shetty, Kahmil Shajihan, Richard E. Neapolitan

**Affiliations:** 1 Department of Biomedical Informatics, University of Pittsburgh School of Medicine, 5607 Baum Blvd, Pittsburgh, PA 15217 USA; 2 Department of Pathology, University of Pittsburgh and Pittsburgh VA Health System, Pittsburgh, PA USA; 3 UPMC Hillman Cancer Center, Pittsburgh, PA USA; 4 Division of Hematology/Oncology, University of Pittsburgh School of Medicine, Pittsburgh, PA USA; 5 Department of Preventive Medicine, Northwestern University Feinberg School of Medicine, Chicago, IL USA

**Keywords:** Risk factor, Interaction, Bayesian network, Breast cancer, Metastasis

## Abstract

**Background:**

Even though we have established a few risk factors for *metastatic breast cancer* (*MBC*) through epidemiologic studies, these risk factors have not proven to be effective in predicting an individual’s risk of developing metastasis. Therefore, identifying critical risk factors for MBC continues to be a major research imperative, and one which can lead to advances in breast cancer clinical care. The objective of this research is to leverage Bayesian Networks (BN) and information theory to identify key risk factors for breast cancer metastasis from data.

**Methods:**

We develop the *Markov Blanket and Interactive risk factor Learner* (*MBIL*) algorithm, which learns single and interactive risk factors having a direct influence on a patient’s outcome. We evaluate the effectiveness of MBIL using simulated datasets, and compare MBIL with the BN learning algorithms *Fast Greedy Search* (*FGS*), *PC algorithm* (*PC*), and *CPC algorithm* (*CPC*). We apply MBIL to learn risk factors for 5 year breast cancer metastasis using a clinical dataset we curated. We evaluate the learned risk factors by consulting with breast cancer experts and literature. We further evaluate the effectiveness of MBIL at learning risk factors for breast cancer metastasis by comparing it to the BN learning algorithms *Necessary Path Conditio*n (*NPC*) and *Greedy Equivalent Search* (*GES*).

**Results:**

The averages of the Jaccard index for the simulated datasets containing 2000 records were 0.705, 0.272, 0.228, and 0.147 for MBIL, FGS, PC, and CPC respectively. MBIL, NPC, and GES all learned that *grade* and *lymph_nodes_positive* are direct risk factors for 5 year metastasis. Only MBIL and NPC found that *surgical_margins* is a direct risk factor. Only NPC found that *invasive* is a direct risk factor. MBIL learned that *HER2* and *ER* interact to directly affect 5 year metastasis. Neither GES nor NPC learned that *HER2* and *ER* are direct risk factors.

**Discussion:**

The results involving simulated datasets indicated that MBIL can learn direct risk factors substantially better than standard Bayesian network learning algorithms. An application of MBIL to a real breast cancer dataset identified both single and interactive risk factors that directly influence breast cancer metastasis, which can be investigated further.

## Background

Breast cancer is one of the leading causes of cancer death in US women [1, 2]. It is estimated that 40,920 US women will die from breast cancer in 2018 [3]. Breast cancer is also one of the main causes of cancer related death in women globally, and it is estimated that without major changes in prevention or treatment, 846,241 women will die from breast cancer worldwide in 2035 [4]. Women do not die of breast cancer confined to the breast or draining lymph nodes; rather, they die mainly due to metastasis, a condition in which cancer spreads to other vital organs such as the lung and brain. *Metastatic breast cancer* (*MBC*) is the cause of over 90% of breast cancer related deaths [5, 6] and remains a largely incurable disease. Although most newly diagnosed breast cancer cases are not metastatic, all patients are at risk of developing metastatic cancer in the future, even if they are free of cancer for years after the initial treatment.

Being able to identify the key direct risk factors for MBC (i.e. risk factors that affect MBC directly rather than just being associative) is important because 1) we can target therapy towards those risk factors; and 2) we can use the risk factors to more effectively predict for each individual patient the likelihood of metastatic occurrence, and this prediction can guide treatment plans tailored to a specific patient to prevent metastasis and to help avoid under- or over-treatment [7]. Researchers have established a few risk factors for MBC through epidemiologic studies, but these risk factors have not proven to be effective in predicting an individual’s risk of developing metastasis, and it has not been shown they have a direct influence on MBC. Therefore, identifying direct risk factors for MBC continues to be a major research imperative. Making further progress in this effort will be a key advancement in breast cancer clinical care, and can significantly improve patient outcomes, and reduce healthcare costs at large.

It is commonplace for medical researchers to obtain retrospective data on patient features and outcomes, and then to analyze the association of possible risk factors with those outcomes. Relative to breast cancer, Curtis et al. [8] developed the METABRIC dataset, which contains clinical and genomic data, along with breast cancer death results, on 1992 breast cancer patients. Zeng et al. [9] analyzed these data and found that *P53_mutation_status, HER2_status, PR_category, ER_category, stage, lymph_nodes_positive, grade, tumor_size, age*, and *chemotherapy* all are highly correlated with 5 year breast cancer death (*p*-value < 10^− 6^). A difficulty with simply identifying risk factors through such correlation analyses is that a risk factor can be correlated with a disease without having any direct or even indirect effect on the disease. For example, menopause status and age might both be associated with MBC. However, menopause status could be a direct risk factor, while age is associated with MBC only because it is correlated with menopause status.

A second difficulty with these correlation analyses is that they usually do not investigate or identify interacting risk factors. An *interaction* is the phenomenon where the predictive effect of one variable is dependent on the presence of one or more other variables. One example of an interaction is genetic epistasis, which describes the situation where genetic mutations have different effects in combination than they do individually. These discrete interactions typically cannot be represented in a regression equation. Indeed they often have little or no marginal effects. So methods have been developed for learning such interactions without making strong mathematical assumptions. These matters are discussed in detail in [9]. Such discrete interactions can also exist in other contexts such as between mRNA and microRNA [10–12], among signal transduction pathways [13], and among risk factors for disease. A pure (no marginal effects) or near-pure interaction is often not-identifiable unless we purposely search for it using a special score measurement. Although Bayesian network algorithms can discover direct risk factors (features that directly affect the outcome of interest), they cannot in general discover interactions with little marginal effects because they assume the composition property, which states that if variable *X* and set of variables *S* are conditionally independent given a set of variables *T*, then there is a variable *Y* in *S* such that *X* and *Y* are conditionally independent give, and on the other hand, if a variable *X* and a set of variables *S* are not independent conditional on *T*, then there exists a variable *Y* in *S* such that *X* and *Y* are not independent conditional on *T* [14]. When *T* is the empty set, this property simply states if *X* and *S* are not independent then there is an *Y* in *S* such that *X* and *Y* are not independent. Therefore, at least one variable in *S* much be correlated with *X*. However, if two or more variables interact in some way to affect *Z*, there could be little marginal effect for each variable, and the observed data could easily not satisfy the composition property. Furthermore, if interacting variables have strong marginal effects, the causal learning algorithms do not distinguish them as interactions, but only as individual causes. So, the standard methods do not learn that risk factors that are interacting to influence a target, and do not even discover risk factors that are interacting with little or no marginal effect. An important task then is to learn such interactions from data. A method that does this could be a preliminary step before applying a causal learning algorithm. This paper concerns the development of a new method that does this in the case of discrete variables. We first provide some examples of situations where discrete variables interact.

In this paper we learn single and interactive direct risk factors for MBC using a large-scale breast cancer dataset, which we curate, and a new algorithm which we develop. The algorithm, called the *Markov Blanket and Interactive risk factor Learner* (*MBIL*) algorithm, focuses on detecting both single and interactive risk factors that have a direct influence on a patient’s outcome such as breast cancer metastasis. The algorithm does not merely find correlation, but rather identifies direct interactive risk factors. We first validate the effectiveness of MBIL using simulated data. We then use MBIL to learn the direct risk factors for 5 year breast cancer metastasis from our *Lynn Sage Datasets* (*LSDS*).

## Methods

Since the new methodology we develop utilizes Bayesian networks and learning interactive risk factors using information theory, we first briefly review these.

### Bayesian networks and the Markov blanket

*A Bayesian network* (*BN*) consists of a *directed acyclic graph* (*DAG*), whose nodeset contains random variables, and the conditional probability distribution of every variable in the network given each set of values of its parents [14–18]. The edges in *E* represent direct probabilistic dependencies. *The Markov blanket* of a given target node *T* in a Bayesian network is the set of nodes *M* such that *T* is probabilistically independent of all other nodes in the network conditional on the nodes in *M*. If we know the value of the nodes in *M*, finding out the values of other nodes cannot change the conditional probability of *T*.

**Fig. 1:**
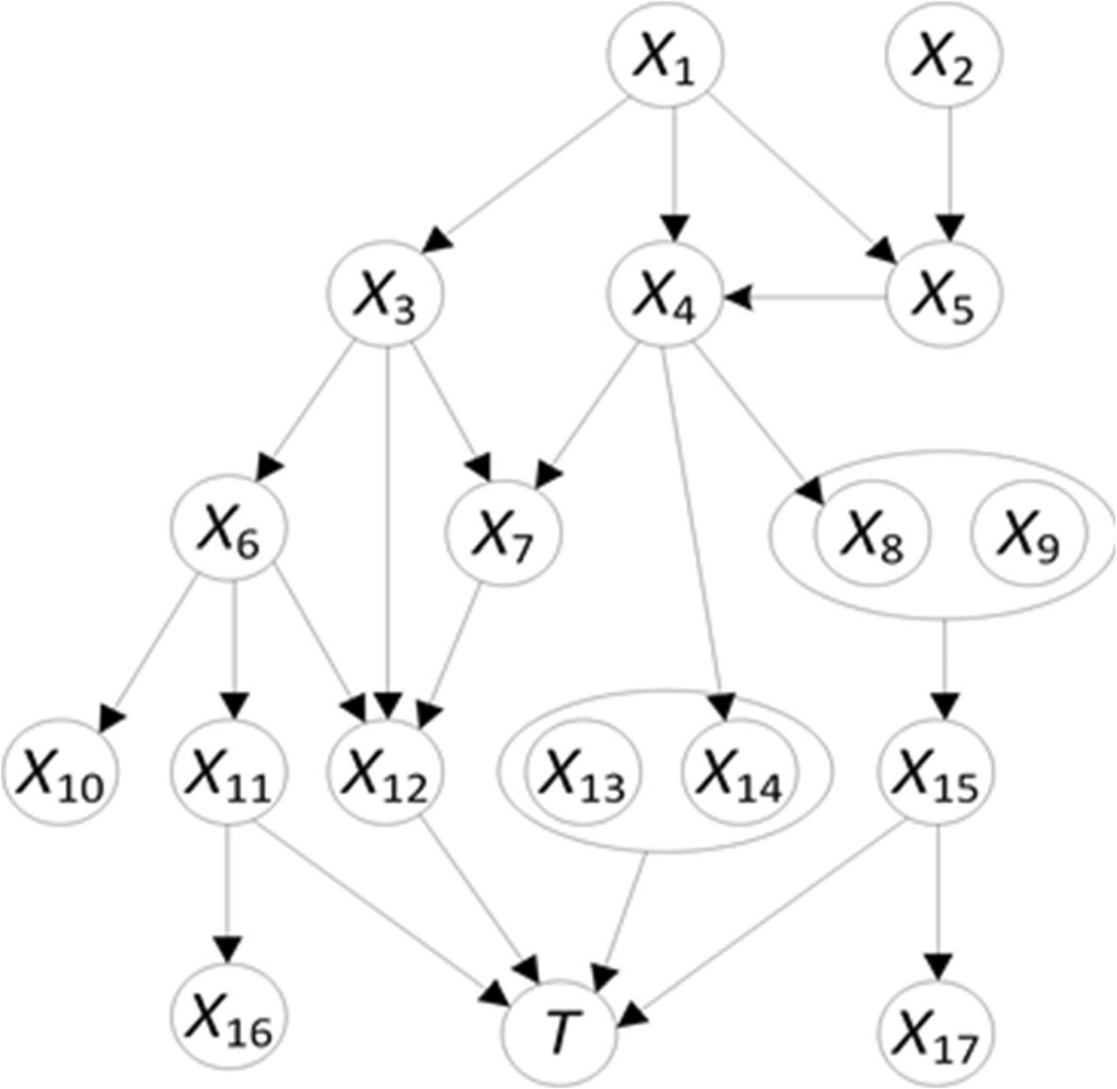
A BN model demonstrating the dependency and independency relationships among variables. The Markov Blanket of *T* consists of nodes *X*_11_, *X*_12_, *X*_13_, *X*_14_ and *X*_15_. These nodes are the direct risk factors and separate *T* from the influence of the noisy predictors *X*_1_-*X*_10_, *X*_16_, and *X*_17_

In general, the Markov blanket of *T* consists of all parents of *T*, children of *T*, and parents of children of *T* [14]. If *T* is a leaf (a node with no children), then the Markov blanket consists only of the parents of *T*, which are the *direct risk factors* for *T* if *T* is a disease. Figure 1 shows a BN DAG model. Since *T* is a leaf in that model, the Markov blanket of *T* consists of is parents, namely nodes *X*_11_-*X*_15_.

**Fig. 2:**
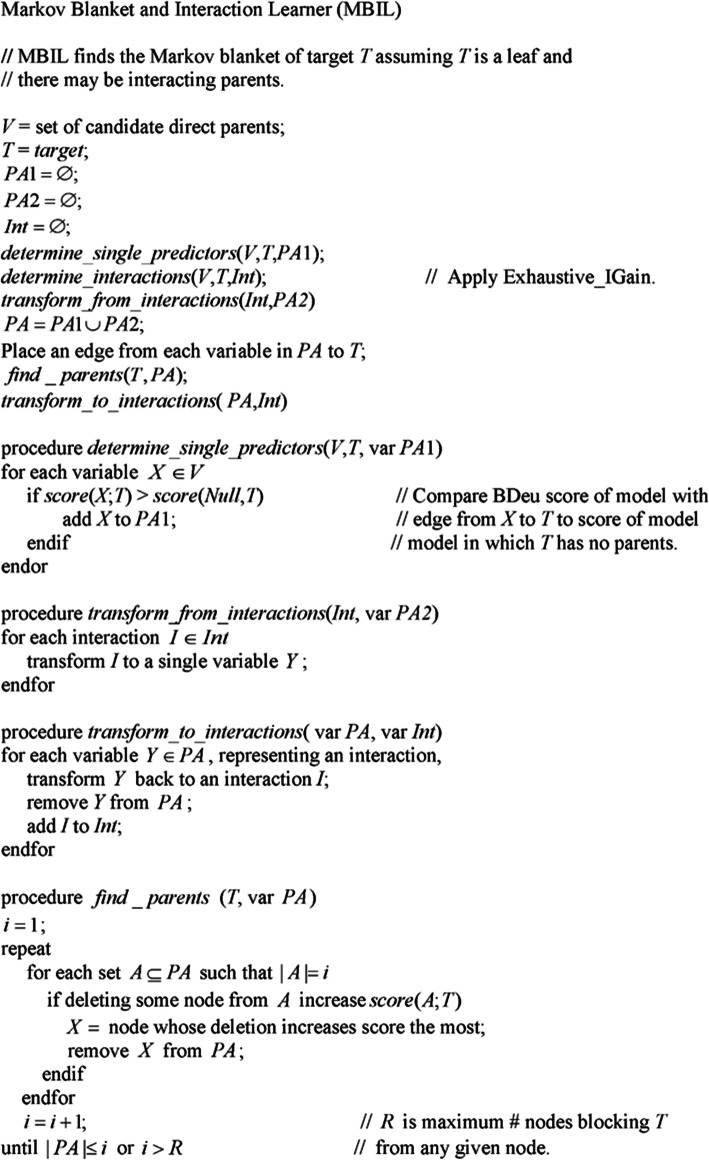
The MBIL algorithm

Knowledge of the direct risk factors in the Markov blanket of a leaf target node *T* such as MBC is valuable in clinical research in two ways. First, we can target therapy towards these direct risk factors rather than risk factors which are merely correlated with MBC and might not even affect MBC. In the network in Fig. 1*X*_17_ is an example of a variable that is correlated with *T* but has no effect on *T*, and therefore should not be targeted. Second, without knowing the BN DAG model, nodes *X*_1_-*X*_10_*X*_16_, and *X*_17_ would all be learned as predictive risk factors due to the fact that they are correlated with *T* through the nodes in the Markov blanket. However, if we can identify the Markov blanket and know the values of the nodes in it, we will have blocked the connections between *T* and the other nodes. So, these *noisy* predictors can be completely removed from a prediction model, which should improve prediction performance.

Learning a BN [14, 19, 20] from data concerns learning both the parameters and the structure (called a *DAG model*). In the score-based structure-learning approach, a score is used to measure how well a DAG represents the data. The *Bayesian score* [21] is the probability of the data given the DAG. A popular variant of this score is the *Bayesian Dirichlet equivalent uniform* (*BDeu*) score, which uses a parameter alpha to represent a prior equivalent sample size [20]. The BN model selection problem is NP-hard [22]. So, heuristic search algorithms are used [14].

### Information theory and learning interactive risk factors

As noted earlier, risk factors may interact to affect disease. For example, in Fig. 1*X*_13_ and *X*_14_ may have a joint effect on *T*, while each has no marginal effect on *T*. A joint predictor like this is often ignored and missed by standard BN structure learning [9, 23]. We previously designed a new information theory-based score to measure the strength of an interaction and applied it to interaction learning. Next we discuss information theory and that score.

Information theory is the discipline that deals with the quantification and communication of information. If *T* is a discrete random variable with *m* alternatives, we define the *entropy H*(*T*) as follows:

$$ H(T)=-\sum \limits_{i=1}^mP\left({t}_i\right){\log}_2P\left({t}_i\right) $$

Shannon [24] showed that if we repeat *n* trials of the experiment having outcome *T*, then the entropy *H*(*T*) is the limit as *n* → ∞ of the expected value of the number of bits needed to report the outcome of each trial of the experiment. Entropy is a measure of our uncertainty in the value of *T* since, as entropy increases, on the average it takes more bits to resolve our uncertainty. The conditional entropy of *T* given *X* is the expected value of the entropy of *T* conditional on *X*. It is defined as follows (where *X* has *k* alternatives):

$$ H\left(T|X\right)=\sum \limits_{j=1}^kH\left(T|{x}_j\right)P\left({x}_j\right). $$

By learning the value of *X*, we can reduce our uncertainty in *T*. The *information gain* (*IG*) of *T* relative to *X* is defined to be the expected reduction in the entropy of *T* conditional on *X*:

$$ IG\left(T;X\right)=H(T)-H\left(T/X\right) $$

The notation *IG*(*T*;*A*), where *A* is set, denotes the information gain based on the joint distribution of all variables in *A*.

Although genetic epistasis serves as a good example of a discrete interaction and intuitively we understand what is meant by an interaction, a formal definition did not exist until Jiang et al. [23] and Zeng et al. [9] developed the interaction strength to both define an interaction and measure its strength. If we have a target variable *T* and two sets of variables *A* and *B*, we define the *interaction strength* (*IS*) of a set of variables *M* relative to a target *T* as follows:

$$ IS\left(T;M\right)=\underset{A\subset M}{\min}\frac{IG\left(T;M\right)- IG\left(T;M-A\right)- IG\left(T;A\right)}{IG\left(T;M\right)} $$

Since information gain (*IG*) is nonnegative, it is straightforward that *IS*(*T*;*M*) ≤ 1. If the variables in *M* are causing *T* with no marginal effects, then *IS* is 1. We would consider this a very strong interaction. When the *IS* is small, the increase in *IG* obtained by considering the variables in *M* together is small compared to considering them separately. We would consider this a weak interaction or no interaction at all.

Jiang et al. [23] show that if the variables in *M* are independent causes of *T*, then

$$ IS\left(T;M-A,A\right)\ge 0 $$

So, in situations we often investigate, the *IS* is between 0 and 1, and therefore satisfies the notion of a fuzzy set [25], where the greater the value of the *IS* the greater membership the set has in the fuzzy set of interactions. Situations in which the *IS* can be negative are discussed in [23].

Zeng et al. [9] developed Exhaustive_IGain, which does an exhaustive search to learn interactions from low-dimensional data. The algorithm determines whether every set of variables *M* is an interaction by checking whether the *IS* exceeds a threshold *t*. If this condition is satisfied, *M* is considered an interaction. They applied Exhaustive_IGain to the METABRIC dataset [1] to investigate how clinical variables might interact to affect breast cancer survival. They found that *hormone_therapy* and *menopausal_status* interact to affect 10 year breast cancer death, but neither *hormone_therapy* nor *menopausal_status* are highly correlated with 10 year breast cancer death alone. A consultation with oncologist Dr. Adam Brufsky revealed that hormone therapy is more effective in post-menopausal women [26].

Jiang et al. [23] developed MBS_IGain, which uses the same strategy as Exhaustive_IGain, but learns interactions from high-dimensional data using a heuristic search. They compared MBS_IGain to 7 other methods using 100 simulated datasets, each based on 5 interactions. MBS_IGain decidedly performed best.

The interactive risk factors we learn using the IGain algorithms may not have a direct influence on the target. That is, they might interact to have an effect on a feature that directly affects the target, or, in the Bayesian network modeling the probabilistic relationships among the variables, they might even be a descendent of a node influencing the target. To identify the direct risk factors of a target we need an algorithm that learns the direct risk factors, including interactive risk factors. We develop such an algorithm next.

### MBIL algorithm

Next we present the new *Markov Blanket and Interactive risk factor Learner* (*MBIL*) algorithm. Figure 2 shows the algorithm, which proceeds as follows:

First, procedure *determine_single_predictors* learns the set *PA*1 of single-variable risk factors of a target *T*. The procedure does this by determining whether the BDeu score of the model in which the variable has an edge to *T* is greater than the BDeu score of the model in which *T* has no parents. Then procedure *determine_interactions* uses Exhaustive_IGain to learn interactive risk factors of *T*. Next procedure *transform_from_interactions* collapses each interaction to a single variable. The set of these new single variables is *PA*2, and the set *PA* = *PA*1∪*PA*2 is the set of all candidate direct risk factors of *T*. Procedure *find_parents* then removes indirect risk factors of *T* from *PA*. It does this as follows. When *i* = 1, the procedure checks whether each one parent model has a lower score than the model with no parents. If it does, the variable in the model is not a direct risk factor and is removed from *PA*. When *i* = 2 it checks, for each two parent models, whether removing one of the variables in the model increases the score of the model. If so, *T* is independent of the removed variable given the other variable, and so the removed variable is not a direct risk factor and is removed from *PA*. Next when *i* = 3 the algorithm checks, for each three parent model, whether removing one of the variables in the model increases the score of the model. If so, *T* is independent of the removed variable given the other two variables, and so the removed variable is not a direct risk factor and is removed from *PA*. The procedure continues in this fashion until *i* equals the size of *PA* or some maximum number *R* of Markov blanket variables is reached. Finally, procedure *transform_to_interactions* transforms the variables representing interactions back to interactions.

Note that the algorithm does not merely find correlation, but rather identifies direct interactive risk factors (features that directly influence the outcome of interest).

The MBIL algorithm has the following parameters:

$$ {\displaystyle \begin{array}{l}\alpha :\mathrm{The}\ \mathrm{value}\ \mathrm{of}\ \upalpha\ \mathrm{in}\ \mathrm{the}\ \mathrm{BDeu}\ \mathrm{score}.\\ {}R1:\mathrm{The} \operatorname {var}\mathrm{iable}\ R\ \mathrm{in}\ \mathrm{procedure}\ \mathrm{determine}\_\mathrm{interactions}\ \left(\mathrm{Exhaustive}\_\mathrm{IGain}\right),\mathrm{which}\ \mathrm{is}\ \mathrm{the}\ \mathrm{maximum}\ \mathrm{size}\ \mathrm{of}\ \mathrm{an}\ \mathrm{in}\mathrm{teraction}.\\ {}R2:\mathrm{The}\ \mathrm{variable}\ R\ \mathrm{in}\ \mathrm{procedure}\ \mathrm{prune}\_\mathrm{nodes},\mathrm{which}\ \mathrm{is}\ \mathrm{the}\ \mathrm{assumed}\ \mathrm{largest}\ \mathrm{number}\ \mathrm{of}\ \mathrm{variable}\mathrm{s}\ \mathrm{in}\ \mathrm{the}\ \mathrm{Markov}\ \mathrm{blanket}.\\ {}t:\mathrm{The}\ \mathrm{threshold}\ \mathrm{in}\ \mathrm{Exhaustive}\_\mathrm{IGain},\mathrm{which}\ \mathrm{is}\ \mathrm{the}\ \mathrm{minimum}\  IS\ \mathrm{needed}\ \mathrm{to}\ \mathrm{be}\ \mathrm{an}\ \mathrm{in}\mathrm{teraction}.\end{array}} $$

### Experiments with simulated datasets

Before applying MBIL to learn risk factors for MBC, we validated that it is effective at this task using simulated datasets.

### Simulated dataset development

We developed six BNs called *Pure_Weak*, *Pure_Strong*, Marginal_Weak_Weak, *Marginal_Weak_Strong*, *Marginal_Strong_Weak*, and *Marginal_Strong_Strong*, which were used to generate the simulated datasets. Next we discuss how each BN was developed.

**Fig. 3:**
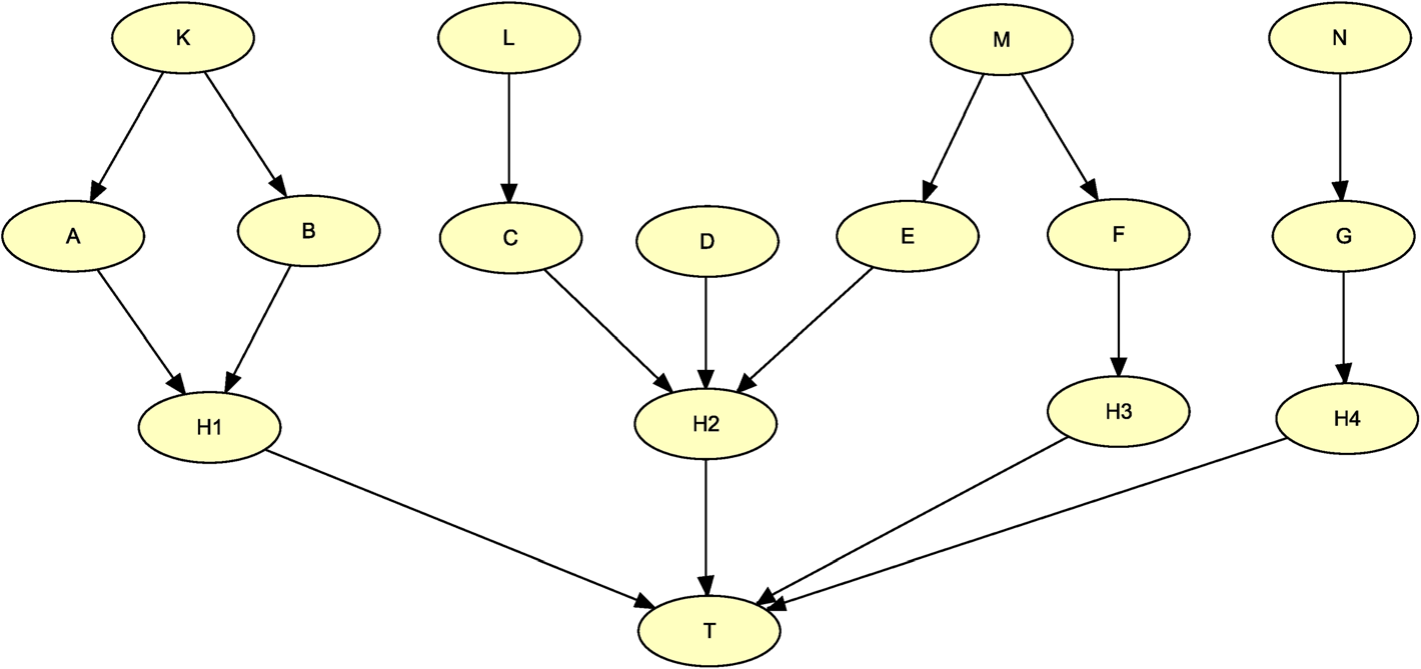
The Bayesian network structure used to generate the simulated datasets

All BNs were generated using the BN DAG model in Fig. 3, each with its own values of the parameters. The variables *A* and *B* are involved in a two-direct risk factor interaction, and this is implemented using the hidden variable *H*1. The variables *C*, *D*, and *E* are involved in a three-direct risk factor interaction, and this is implemented using the hidden variable *H*2. The variables *F* and *G* are each stand-alone binary direct risk factors and are implemented using *H*3 and *H*4. The effects of the hidden variables *H*1, *H*2, *H*3, and *H*4 are combined using the Noisy-OR model [14, 16]. Next we describe how each interaction was generated for each BN.

**Fig. 4:**
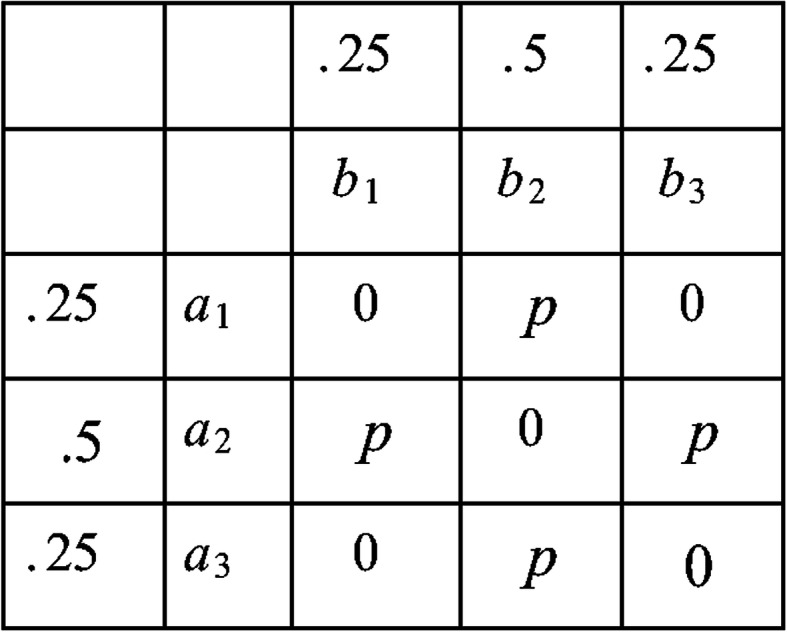
The relationships used to generate interaction (*A*,*B*) in Fig. 3. (The number next to each variable value shows the fraction of occurrence of that value in the population, and the entries in the table show the probability that *H*1 equals 1 given each combination of values of *A* and *B*)

The interaction between *A* and *B* was based on the relationships in Fig. 4, which provides a pure interaction. Variables *A* and *B* are both trinary predictors of the binary target *H*1, which has values 0 and 1. The number next to each variable value shows the fraction of occurrence of that value in the population, and the entries in the table show the probability *H*1 equals 1 given each combination of values of *A* and *B*. For example,

$$ P\left(H1=1\ |A={a}_1,B={b}_2\right)=p. $$

Next we shows that variable *A* has no marginal effect. We have

$$ {\displaystyle \begin{array}{c}P\left(H1=1|A={a}_1\right)=0.0\times 0.25+p\times 0.5+0.0\times 0.25=0.5p\\ {}P\left(H1=1|A={a}_2\right)=p\times 0.25+0\times 0.5+p\times 0.25=0.5p\\ {}P\left(H1=1|A={a}_3\right)=0.0\times 0.25+p\times 0.5+0.0\times 0.25=0.5p\end{array}} $$

Similarly, variable *B* has no marginal effect.

Urbanowicz et al. [27] created GAMETES, which is a software package for generating pure, strict epistatic models with random architectures. The software allows the user to specify the *heritability* and the *minor allele frequency*. We used GAMETES to generate the interaction between *C*, *D*, and *E*.

For the BN called *Pure_Weak*, interaction (*A*,*B*) was generated with *p* = 0.3 in Fig. 3; interaction (*C*,*D*,*E*) was generated with 0.3 heritability and 0.3 minor allele frequency in GAMETES; and the *P*(*H*3 = 1|*F* = 1) and *P*(*H*4 = 1|*G* = 1) (called the strengths with which binary risk factors *F* and *G* influence *T*) were set to 0.3, while *P*(*H*3 = 1|*F* = 0) and *P*(*H*4 = 1|*G* = 0) were set to 0. For the BN called *Pure_Strong*, interaction (*A*,*B*) was generated with *p* = 0.8 in Fig. 3; interaction (*C*,*D*,*E*) was generated with 0.5 heritability and 0.4 minor allele frequency in GAMETES; and the strengths for *F* and *G* were set to 0.8.

Interactions in the BN called *Marginal_Weak_Weak* were generated using the same relationships as in the BN called *Pure_Weak*, except the variables involved in interactions were allowed to have weak marginal effects. Interactions in the BN called *Marginal_Weak_Strong* were generated using the same relationships as in the BN called *Pure_Weak* except the variables involved in interactions were allowed to have strong marginal effects. In the same way the interactions in the BNs called *Marginal_Strong_Weak* and *Marginal_Strong_Strong* were based on the interactions in the BN model called *Pure_Strong*.

Using each of the 6 BNs, we generated 20 datasets with 2000 records, making a total of 120 datasets. We call the set of all these datasets the *Learn_Datasets*. Using each of the 6 BNs, we generated another 20 datasets with 2000 records and 20 datasets with 10,000 records, making a total of 240 datasets. We call the set of these 240 datasets the *Test_Datasets*.

### Simulated dataset analysis

Using the simulated datasets, we compared MBIL to three standard BN learning algorithms, namely *Fast Greedy Search* (*FGS*), the *PC algorithm* (*PC*), and the *CPC algorithm* (*CPC*). We used our own implementation of MBIL, and the implementations of FGS, PC, and CPC in Tetrad [18]. The target was forced to be a leaf node in the models learned by FGS, PC, and CPC.

We applied each method to the *Learn_Datasets* to learn direct risk factors for target *T*. For MBIL we set *R*_1_ = *R*_2_ = 5. We then used *t* = 0.1 and *t* = 0.2 combined with the values 1, 120, 240, and 480 of α. FGS has two parameters, *SMP* and *STP*. We used the following combinations of these parameters: *SMP* = 1, *STP* = 1; *SMP* = 1,*STP* = 2; *SMP* = 2,*STP* = 1; *SMP* = 2,*STP* = 2. PC and CPC each have one parameter α; we used α = 0.01, α = 0.05, α = 0.1, and α = 0.2, α = 0.3.

The *Jaccard index* for two sets *V* and *W* is defines as follows:

$$ Jaccard\left(A,B\right)=\frac{\#\left(A\cap B\right)}{\#\left(A\cup B\right)} $$

The Jaccard index equals 1 if the two sets are identical and equals 0 if their intersection is empty. After applying each of the 4 methods with each of its parameter settings to all 120 of the Learn_Datasets, we computed the Jaccard index of the set of true direct risk factors *V* = {*A,B,C,D,E,F,G*} and the set of learned direct risk factors *W* for each application. Then for each parameter setting, we computed the average Jaccard index. For each of the 4 methods we chose the parameter setting which gave the highest average Jaccard index. The best parameter setting were as follows:

$$ {\displaystyle \begin{array}{l}\mathrm{MBIL}:t=0.2,\alpha =480\\ {}\mathrm{FGS}: SMP=2, STP=2\\ {}\mathrm{PC}:\alpha =0.2\\ {}\mathrm{CPC}:\alpha =0.2.\end{array}} $$

Finally, we applied each of the 4 methods with its best parameter settings to the *Test_Datasets*, and for each method we computed the average Jaccard index separately for the datasets containing 2000 records and for the datasets containing 10,000 records.

### Applying MBIL to learn direct risk factors for MBC

#### Dataset Curation

The *Lynn Sage database* (*LSDB*) contains information about patients who came to the Lynn Sage Comprehensive Breast Center at Northwestern Memorial Hospital for care. The *Northwestern Medicine Enterprise Data Warehouse* (*NMEDW*) is a joint initiative across the Northwestern University Feinberg School of Medicine and Northwestern Memorial HealthCare, which maintains comprehensive data obtain from *electronic health records* (*EHR*). Using the LSDB and the NMEDW, we curated a dataset called the *Lynn Sage Dataset* (*LSDS*). The LSDS is comprised of records on 6726 breast cancer patients, which span 03/02/1990 to 07/28/2015. The dataset contains 32 patient features such as *HER2* status and *grade*, outcome variables including *distant metastasis*, l*ocal recurrence*, and o*verall survival*, along with the number of days after initial consultation these events occurred, and treatment variables including *breast/chest wall radiation, nodal field radiation, alkylating agents, anthracyclines, antimetabolites, anti-tubulin, neoadjuvant therapy, gonadotrophin releasing hormone agonists, targeted therapy, HER2 inhibitors, anastrozole, tamoxifen, and letrozole.*

Our goal in this study was to find the direct risk factors for 5 year breast cancer metastasis. For the purpose of this study, we eliminated all variables that were redundant or composites of other variables (e.g. *stage*), and all variables that have 1/3 or more missing values. So, the candidate risk factors included in the study are the ones shown in Table 1. We assigned the value *yes* to metastasis if the patient metastasized within 5 years of initial diagnosis, the value *no* to metastasis if it was known that the patient did not metastasize within 5 years, and the value NULL to metastasis if the patient discontinued follow-up within the first 5 years and had not metastasized. The value NULL was also assigned to all missing data fields in all variables. Missing data (NULL values) were filled in using the *nearest neighbor* imputation algorithm.

**Table 1:** The candidate direct risk factors in the LSDS that are analyzed in the study presented here

Candidate Risk Factor	Description	Values
*race*	race of patient	white, black, Asian
*age_at_diagnosis*	age at diagnosis of the disease	0–40, 40–55, 55–69, > 69
*menopausal_status*	inferred menopausal status	pre, post
*size*	size of tumor in mm	0–32, 32–70, > 70
*lymph_nodes_positive*	number of positive lymph nodes	0, 1–8 > 8
*lymph_nodes_removed*	number of lymph nodes removed	0–11, 12–22, > 22
*lymph_node_status*	patient had any positive lymph nodes	neg,pos
*grade*	grade of disease	1, 2, 3
*invasive*	whether tumor is invasive	yes,no
*histology*	tumor histology	lobular, duct
*ER*	estrogen receptor expression	neg, pos
*PR*	progesterone receptor expression	neg, pos
*HER2*	HER2 expression	neg, pos
*P53*	whether P53 is mutated	neg, pos
*surgical_margins*	whether residual tumor	res. Tumor, no res. Tumor, no primary site surgery

We used MBIL, the constraint-based Bayesian network learning algorithm *Necessary Path Conditio*n (*NPC*, and the score-based Bayesian network learning algorithm *Greedy Equivalent Search* (*GES*) with the *Bayesian information criterion* (*BIC*) score to learn the direct risk factors for 5 year breast cancer metastasis. We used our own implementation of MBIL and the implementations of NPC and GES in the Hugin package (https://www.hugin.com/). The variable *metastasis* was forced to be a leaf node in the networks learned by NPC and GES.

## Results

### Simulated data

Table 2 shows the average Jaccard indices for the *Test_Datasets* containing 2000 records. As would be expected, in general the performance of all methods increases as we increase the strength of the interactive effect and the strength of the marginal effects. MBIL substantially outperformed the other methods overall and for every dataset type. Oddly, CPC performs very poorly with the weak datasets, but performs the best of the other methods for the *Marginal_Strong_Strong* datasets.

**Table 2:** Average Jaccard index results for 120 datasets containing 2000 records

Dataset	MBIL	FGS	PC	CPC
*Pure_Weak*	0.509	0.254	0.186	0.033
*Marginal_Weak_Weak*	0.631	0.250	0.171	0.050
*Marginal_Weak_Strong*	0.650	0.257	0.214	0.043
*Pure_Strong*	0.700	0.286	0.239	0.154
*Marginal_Strong_Weak*	0.850	0.286	0.254	0.232
*Marginal_Strong_Strong*	0.890	0.300	0.304	0.368
All Datasets	0.705	0.272	0.228	0.147

Table 3 shows the average Jaccard indices for the *Test_Datasets* containing 10,000 records. Again, MBIL substantially outperforms the other methods. In fact, its detection performance is close to perfect when the causal strength is strong, even when there are no marginal effects. This results indicates that, with a sufficient amount of data, MBIL can accurately detect all pure interactions with few false positives when the causal strength is strong. It is somewhat odd that, in the case of the weak interactions, the performance of MBIL degrades when we have marginal effects. This could be due to the parent of a variable, which is involved in an interaction, being detected as a stand-alone risk factor owing to the marginal effect of the variable. A visual inspection of some of the results indicated this.

**Table 3:** Average Jaccard index results for 120 datasets containing 10,000 records

Dataset	MBIL	FGS	PC	CPC
*Pure_Weak*	0.707	0.286	0.25	0.000
*Marginal_Weak_Weak*	0.651	0.286	0.271	0.186
*Marginal_Weak_Strong*	0.645	0.321	0.339	0.336
*Pure_Strong*	0.989	0.286	0.213	0.275
*Marginal_Strong_Weak*	1.000	0.321	0.414	0.664
*Marginal_Strong_Strong*	1.000	0.568	0.611	0.811
All Datasets	0.832	0.345	0.350	0.379

Our results using simulated datasets indicate MBIL is more effective than current methods at learning direct risk factors. So, we next applied it to learn direct risk factors for 5 year MBC.

### Application of MBIL to the LSDS breast Cancer dataset

Table 4 shows the direct risk factors learned by MBIL, NPC, and GES from the LSDS. All three methods learned that *grade* and *lymph_nodes_positive* are direct risk factors. It is plausible that each of these variables would have a direct influence on metastasis. Only MBIL and NPC found that *surgical_margins* is a direct risk factor. It is also plausible that this variable would have a direct influence on metastasis. Only NPC found that i*nvasive* is a direct risk factor of metastasis. An inspection of the entire network learned by GES reveals that GES determined that *invasive* and *surgical_margins* are independent of metastasis given *lymph_nodes_positive* according to that network. These conditional independencies are also plausible.

**Table 4:** Direct risk factors for 5 year breast cancer metastasis learned by MBIL, NPC, and GES. (IS: *interaction strength* as described on page 6; NA: not applicable because IS requires at least two variables)

Method	Cause	IS
MBIL	*grade*	NA
	*lymph_nodes_positive*	NA
	*surgical_margins*	NA
	*HER2, ER*	0.1297
NPC	*grade*	NA
	*lymph_nodes_positive*	NA
	*surgical_margins*	NA
	*invasive*	NA
GES	*grade*	NA
	*lymph_nodes_positive*	NA

Most notable is that MBIL learned that *HER2* and *ER* interact to directly affect metastasis. This interaction is well-known [28]. Neither GES nor NPC learned that *HER2* and *ER* are direct risk factors. An inspection of the entire networks learned by these algorithms reveals that each of them discovered that HER2 and *ER* are independent of metastasis given numerous variables. The effects of HER2 and *ER* on metastasis, when each is taken alone, are too small for them to be recognized as direct risk factors. The interactive strength of 0.1297 (Table 4) for *HER2* and *ER* may not seem that high. However, most variable pairs have interactive strengths about equal to 0. This interaction of hormone receptors and growth factor receptors may be biologically due to physical protein interactions or to the availability of specific treatment against these two markers with either intervention improving survival. In the latter case, this would represent an interaction that is present due to our therapeutic developments and would not be observed in naïve tumors.

Note that our algorithm detects the direct risk factors (features that directly influence the outcome of interest). This means that, according to our data, all other variables are correlated with 5-year metastasis only through their association with our learned direct risk factors. For example, according to a chi-square test, *size* and 5-year metastasis are associated with *p*-value less than 0.00001. However, the MBIL results indicate that this association is through *grade, lymph_nodes_positive, surgical_margins,* and/or *HER2, E*R. It could be that *grade* affects both *size* and 5-year metastasis, and *size* has no effect at all. Or *size* could indirectly affect 5-year metastasis. Regardless, based on our results, we can direct therapy at discovered direct risk factors rather than at variables only known to be correlated.

**Fig. 5:**
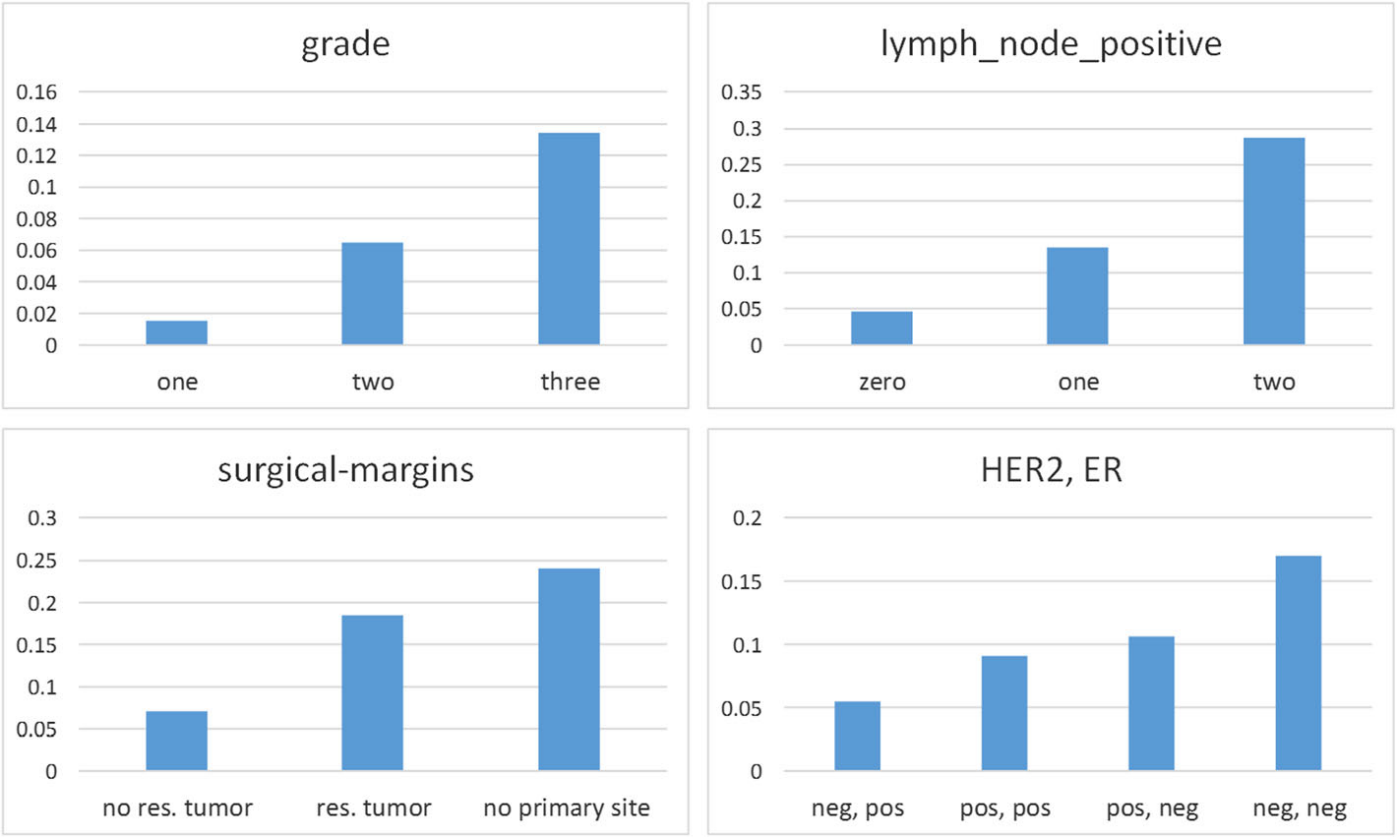
The probabilities of metastasis given the values of the direct risk factors for 5 year breast cancer metastasis learned by MBIL

As shown in Fig. 5, the direct risk factors learned by MBIL have a considerable probabilistic association with 5 year metastasis based on the data in the LSDS. The probabilities for values of *stage*, *lymph_nodes_positive*, and *surgical_margins* are substantial and in the order we would expect. The order for *HER2, ER* are in agreement with the order reported in [29], except we have *HER2+,ER-* and *HER2-,ER-* reversed relative to their order. However, we have 6726 records compared to their 1134 records, and other research indicates triple negative breast cancer (*HER2-,ER-,PR-*) has the worst prognosis [30], in agreement with our results. In addition, while the natural history of *HER+* breast cancer demonstrates greater aggressiveness, the availability of targeted therapy has significantly changed the outcome for the better for these patients [31, 32]..

The probability of 5 year metastasis (based on the LSDS) given the most benign values of all the direct risk factors is 0.015, while the probability of metastasis given the most severe values is 0.468. These results are not shown in the table or figure.

## Discussion

We developed the LSDS and the MBIL algorithm to learn interactive direct risk factors for 5 year MBC. The results of experiments involving simulated data indicated MBIL can learn such direct risk factors significantly better than the standard Bayesian network algorithms FGS, PC, and CPC. We then applied MBIL and the standard algorithms GES and NPC to the real LSDS to learn the direct risk factors for 5 year MBC. The results for MBIL were more cogent than those for the other methods. In particular MBIL learned that *HER2* and *ER* interact to directly affect 5 year breast cancer metastasis, while other methods did not. Their interaction is well-known.

Knowledge of the direct risk factors for metastasis can be used in two ways. First, we can target interventions towards these risk factors. Since *lymph_nodes_positive* is a direct risk factor, there is no point in targeting therapy towards factors which only affect the primary tumor since by this time the nodes are positive or not. However, we can target the micro-metastases in the lymphatic system. If *surgical_margins* are known to be a direct risk factor, surgeons can re-explore the surgery site if there is a residual tumor. Further, if there are either positive margins or lymph nodes, the patient may be candidate for adjuvant therapy for cryptic micrometastases even prior to emergence. However, we can spare the toxic effects for those patients less likely to harbor such hidden tumor cells.

The interaction of *HER2* and *ER* was found to be a direct risk factor, with the combination of both taking negative values having the highest risk. We already have therapy targeting *HER2* positive and *ER* positive. However, there is no targeted therapy in the case where both are negative. Currently, *triple negative breast cancer* (*TNBC*) is consider a candidate for aggressive but non-specific adjuvant therapies (chemotherapy and radiation therapy) even in the absence of evidence of metastasis. These finding herein, support such an approach based on risk factors. Still our results indicate developing targeted therapies against these cryptic micrometastases would be a promising area of research.

Secondly, if we eliminate the “noisy” predictors and only use the direct risk factors in a metastasis prediction model, we should improve prediction performance and avoid over-fitting. We plan to investigate this conjecture in future research.

Most of our learned risk factors are well-known. However, as discussed near the end of the last section, it is not known that they are the direct risk factors, Furthermore, to our knowledge, we are the first to further verify risk factors systematically from a different aspect, that is, through big data learning. We demonstrated via these results the importance of data-oriented precision medicine; namely a “discovery” that was made through years of cumulative observations and/or experiments can be identified effectively by running software using available passive data. Secondly, we used these known risk factors as gold standards to evaluate the effectiveness of our method. Note that our method is readily reusable, so, it can be used on other cancer datasets once they become available. If our method can identify well-known risk factors, then we would be more confident it can be applied to other dataset to identify unknown risk factors or interactions.

We applied MBIL to one dataset, and obtained meaningful results that are consistent with current knowledge. We need to develop additional EHR-based breast cancer datasets like the LSDS. MBIL can be applied to each of them individually and together via a meta-analysis, enabling us to accurately determine direct risk factors and predict metastasis taking into account all patient information. This would set the stage for immediate tailoring of existing approaches taking into account metastatic probability, and over the longer term, allowing for the development of targeted, ‘smart’ therapies.

## Conclusions

We conclude that the MBIL algorithm is effective at identifying direct risk factors for patient outcomes, and that it has successfully identified likely direct risk factors for 5 year breast cancer metastasis.

## Data Availability

We have submitted our dataset LSDB-5YDM to datadryad.org (DOI 10. 5061/dryad.64964 m0).

## Abbreviations

MBC: Metastatic breast cancer
MBIL: Markov Blanket and Interactive risk factor Learner
FGS: Fast Greedy Search
PC: PC algorithm
CPC: CPC algorithm
NPC: Necessary Path Condition
GES: Greedy Equivalent Search
LSDS: Lynn Sage Datasets
BN: Bayesian network
DAG: Directed acyclic graph
BDeu: Bayesian Dirichlet equivalent uniform
IG: Information gain
IS: Interaction strength
LSDB: Lynn Sage database
NMEDW: Northwestern Medicine Enterprise Data Warehouse
EHR: Electronic health records
BIC: Bayesian information criterion
TNBC: Triple negative breast cancer
